# Hidden Targets in Cancer Immunotherapy: The Potential of “Dark Matter” Neoantigens

**DOI:** 10.3390/vaccines14010104

**Published:** 2026-01-21

**Authors:** Francois Xavier Rwandamuriye, Alec J. Redwood, Jenette Creaney, Bruce W. S. Robinson

**Affiliations:** 1 National Centre for Asbestos Related Diseases, Institute for Respiratory Health, Nedlands, WA 6009, Australia; francois.rwandamuriye@uwa.edu.au (F.X.R.); alec.redwood@uwa.edu.au (A.J.R.); jenette.creaney@uwa.edu.au (J.C.); 2 School of Biomedical Sciences, University of Western Australia, Crawley, WA 6009, Australia; 3 Department of Respiratory Medicine, Sir Charles Gairdner Hospital, Nedlands, WA 6009, Australia; 4 Medical School, University of Western Australia, Crawley, WA 6009, Australia

**Keywords:** neoantigen, vaccine, non-canonical, dark matter, mRNA, peptide, cryptic

## Abstract

The development of cancer immunotherapies has transformed cancer treatment paradigms, yet durable and tumour-specific responses remain elusive for many patients. Neoantigens, immunogenic peptides arising from tumour-specific genomic alterations, have emerged as promising cancer vaccine targets. Early-phase clinical trials using different vaccine platforms, including mRNA, peptide, DNA, and viral vector-based personalised cancer vaccines, have demonstrated the feasibility of targeting neoantigens, with early signals of prolonged survival in some patients. Most current vaccine strategies focus on canonical neoantigens, typically derived from exonic single-nucleotide variants (SNVs) and small insertions/deletions (INDELs), yet this represents only a fraction of the potential neoantigen repertoire. Evidence now shows that non-canonical neoantigens, arising mostly from alternative splicing, intron retention, translation of non-coding RNAs, gene fusions, and retroelement activation, broaden the antigenic landscape, with the potential for increasing tumour specificity and immunogenicity. In this review, we explore the biology of non-canonical neoantigens, the technological advances that now enable their systematic detection, and their potential to inform next-generation personalised cancer vaccines.

## 1. Introduction

The past decade has seen a paradigm shift in oncology, with therapies increasingly designed not only to directly target tumour cells but also to mobilise the immune system for durable tumour control [1]. Immune checkpoint inhibitors (ICIs) [2,3] and adoptive cell therapies [4] have delivered significant benefits for some patients, but many tumours lack sufficient cancer-specific T cells, induce only weak responses, or develop resistance, highlighting the need for strategies that also strengthen and broaden immune engagement [5].

Neoantigens, peptides generated by tumour-specific genomic alterations, are considered to be highly attractive vaccine targets because they are absent from normal tissues and hence escape central tolerance [6]. Early-phase clinical trials of neoantigen-based cancer vaccines have demonstrated that personalised neoantigen cancer vaccines are feasible, safe, and capable of inducing robust antigen-specific T-cell responses with early signs of clinical benefits [7,8,9]. However, most of the focus has been on canonical neoantigens, which are relatively easy to find, but it is now clear from animal and human studies that there are many more non-canonical neoantigens present [10].

## 2. Neoantigen Cancer Vaccines

Current neoantigen vaccine designs typically aim to include 10–40 predicted neoantigens per patient, predominantly canonical epitopes derived from nonsynonymous (ns) SNVs or INDELs within coding regions. These candidates are selected from the hundreds or thousands of mutations identified through sequencing and bioinformatic pipelines [7,8,9,11]. Although spontaneous T-cell responses to individual tumour mutations are generally rare, vaccination consistently enhances the proportion of neoantigens capable of eliciting measurable antigen-specific T-cell responses. Across clinical trials, including peptide, mRNA, and dendritic cell (DC)-based vaccines, approximately 10–60% of vaccine-encoded epitopes have induced CD4^+^ and/or CD8^+^ T-cell responses, with the exact proportion shaped by tumour type, mutation burden, platform, adjuvant formulation, and the stringency of neoantigen selection [7,8,9,11,12,13].

At the same time, ICI therapy provides clear proof that strong antitumour immune responses can be unleashed, even in tumours with low tumour mutation burden (TMB) [14,15,16]. However, despite these clinical effects, the specific antigens recognised by ICI-expanded T cells remain elusive and thus largely undefined. This creates a gap between the therapeutic benefit observed and our understanding of the underlying antigenic targets. This disparity suggests that key targets of effective antitumour immunity may extend beyond canonical SNV/INDEL-derived neoantigens, highlighting the importance of systematically exploring broader classes of tumour antigens with the potential to be more consistently presented and clinically relevant.

Importantly, recent studies demonstrate that tumours also generate antigens through non-canonical processes such as aberrant splicing, intron retention, translation of long non-coding or circular RNA, pseudogene expression, and activations of retroelements [17,18,19,20,21]. These mechanisms create an overlooked pool of “hidden” targets with the potential for greater tumour specificity and immunogenicity. Here, we synthesise the current knowledge on non-canonical antigen biology and highlight how these previously overlooked antigen sources may broaden the therapeutic landscape for cancer vaccines.

## 3. Why Neoantigen-Based Cancer Vaccines Are a Promising Future Cancer Therapy

The reasons neoantigen cancer vaccines have gained rapid momentum are an increasing number of clinical studies demonstrating feasibility, safety, and immunogenic potential across diverse cancers, also revealing the limitations and challenges facing this field [reviewed in [22]]. Recent clinical trials across a spectrum of cancer types and vaccine delivery formats confirm that personalised cancer vaccines can induce polyfunctional T-cell responses, with encouraging signals of clinical benefit (Table 1). However, most published studies to date are small-phase I/II trials not powered for efficacy, and outcomes remain variable [7,9,23].

Encouraging results come from recent trials using mRNA vaccines. In patients with resected high-risk stage III/IV melanoma, the KEYNOTE-942 phase 2b trial investigated the personalised vaccine mRNA-4157, encoding up to 34 neoantigens from exonic nsSNVs and INDELs in combination with pembrolizumab. At 18-month follow-up, patients had improved recurrence-free (79% vs. 62%) and distant metastasis-free survival (92% vs. 77%) compared with pembrolizumab alone, with benefits observed irrespective of PD-L1 status or TMB [8]. Although immune monitoring data were not reported in this phase 2b publication, earlier phase 1 results from KEYNOTE-603 demonstrated that mRNA-4157 induces polyfunctional CD4^+^ and CD8^+^ T-cell responses against ~23–30% of encoded epitopes, including de novo repertoire formation and memory T-cell responses [24].

In the adjuvant setting for pancreatic ductal adenocarcinoma (PDAC), traditionally considered a “cold” tumour, the personalised mRNA vaccine autogene cevumeran, encoding up to 20 neoantigens from exonic nsSNVs and INDELs, induced polyclonal polyfunctional CD8^+^ T-cell responses in 50% of patients, with responders experiencing longer recurrence-free survival (RFS) than non-responders. Vaccine-induced T cells persisted for up to two years after surgery, and responders had a median RFS not reached versus 13.4 months in non-responders [7]. Using longitudinal CloneTrack data, the authors applied standard exponential-decay modelling of individual T-cell clones to estimate their persistence, showing that vaccine-induced CD8^+^ T-cell clones have extended lifespans, with some predicted to persist for many years following priming and booster doses, with an estimated average lifespan of 7.7 years (range 1.5–100 years), supporting the generation of long-lived durable effector T cells [25]. When examined at the individual-epitope level, autogene cevumeran elicited responses to ~11% of encoded neoantigens by a high-stringency ex vivo assay, rising to 24% among clinical responders, probably related to the restricted canonical neoantigen repertoire characteristic of PDAC rather than a limitation of the vaccine platform. In a separate trial in advanced solid tumours, autogene cevumeran also demonstrated immunogenicity, with CD4^+^ and CD8^+^ T-cell responses detected in 71% of evaluable patients. In some cases, vaccine-specific CD8^+^ T cells comprised up to 23% of circulating lymphocytes and infiltrated tumours [26].

Earlier evidence for personalised neoantigen vaccination came from peptide-based approaches, which were first established in melanoma. In the original NeoVax phase I trial, a personalised synthetic long-peptide vaccine induced broad neoantigen-specific immune responses. Approximately 60% of long peptides (58/97 peptides) elicited CD4^+^ T-cell responses, whereas ~16–20% (15/97 peptides) of predicted class I epitopes generated CD8^+^ T-cell responses, although many CD8^+^ responses were detectable only after in vitro expansion [12].

Clinically, eight out of ten patients remained recurrence-free at a median follow-up of 25 months, and, among the two patients who later relapsed, both responded to subsequent anti-PD-1 therapy, consistent with vaccine-primed T cells enhancing responsiveness to immune checkpoint blockade [12]. A later study by the same group further characterised the quality and breadth of responses, showing that ~10% of immunising long peptides elicited both CD4^+^ and CD8^+^ T-cell responses, and that 20% induced ex vivo CD4^+^ T-cell responses. At the individual-patient level, response breadth was heterogeneous, with recognition of 2–25 assay peptides across CD4^+^ and CD8^+^ T cells [9].

Building on this work, Blass et al. developed NeoVax-MI, a multi-adjuvant personalised peptide vaccine engineered to enhance CD8^+^ T-cell priming. The combined use of Montanide, poly-ICLC, local low-dose ipilimumab, and systemic nivolumab with a cancer vaccine containing 12–20 peptides induced markedly stronger CD8^+^ T-cell responses than earlier synthetic long peptide (SLP) approaches [11]. Vaccine-specific CD8^+^ T-cell clones were detected in eight of nine evaluable patients, reached up to ~20% of circulating CD8^+^ T cells, infiltrated tumours, and displayed polyfunctional memory phenotypes. Notably, 63% of immunising long peptides elicited ex vivo T-cell responses, a substantial increase compared with earlier NeoVax formulations, and these enhanced responses correlated with tumour regression. Together, these studies demonstrate that peptide-based neoantigen vaccination can elicit broad and durable T-cell immunity, and that optimisation of prediction pipelines and adjuvant combinations can substantially improve CD8^+^ T-cell response magnitude and breadth.

Beyond mRNA and peptide platforms, other vaccine delivery strategies have also demonstrated promising immunogenicity. A personalised DNA vaccine (GNOS-PV02) encoding up to 40 neoepitopes together with IL-12 and pembrolizumab elicited neoantigen-specific T-cell responses in 91% of evaluable patients with hepatocellular carcinoma and achieved a 31% objective response rate, with greater efficacy when ≥30 antigens were included [27]. In triple-negative breast cancer, a phase I personalised DNA vaccine induced neoantigen-specific T-cell responses in 14 of 18 patients, and 88% of patients remained cancer-free at three years compared with ~50% in historical controls, although these findings remain preliminary due to the small cohort and lack of a comparator arm [28]. Further, a recent phase I trial in metastatic lung cancer using a personalised neoantigen-loaded DC-based vaccine (Neo-DCVac) demonstrated that autologous DCs pulsed with 12–30 neoantigen peptides can reliably prime mutation-specific immunity. Although patients mounted T-cell responses to only 1–5 neoantigens, these responses were strictly mutant-selective and included both CD4^+^ and CD8^+^ T-cell reactivity, illustrating that DC vaccination can expand scarce pre-existing clones and generate de novo responses that may be limited by natural cross-presentation [29].

**Table 1:** Selected recent clinical trials for personalised neoantigen cancer vaccines.

Platform	Name/ Formulation	Number of Neoantigens	Route	Type of Cancer/Setting	Combination	No. Patients	Study ID	T-Cell Responses	Ref.
Peptide	NeoVax: SLP + poly-ICLC	nsSNV, up to 20 neoantigens	SC	Advanced RCC, Resected	ICI	9 patients	Phase 1 NCT02950766	Mostly CD4^+^ T cells, CD8^+^ T cells after expansion	Braun et al. [23]
	NeoVax: SLP + poly-ICLC	nsSNV, up to 20 neoantigens	SC	Advanced melanoma, resected	ICI	8 patients	Phase 1 NCT01970358	Mostly CD4^+^ T cells, CD8^+^ T cells after expansion	Hu et al. [30]
	NeoVax^MI^: SLP + poly-ICLC + montanide	nsSNV, 8–19 neoantigens	SC	Advanced melanoma, resected	ICI	10 patients	Phase 1 PMID: 40645179	CD8^+^ T-cell responses observed in 66.7% of patients	Blass et al. [11]
mRNA	Autogene cevumeran, mRNA- LPX	nsSNV up to 20 neoantigens	IV	Resected PDAC	ICI + Chemotherapy	16 patients	Phase 1 NCT04161755	Mostly CD4^+^ T cells, low frequency CD8^+^ T cells	Rojas et al. [7] Sethna et al. [25]
	mRNA-4157, mRNA-LNP	nsSNVs, up to 34 neoantigens	IM	Resected stage IIIB–IV melanoma	ICI	157 patients	Phase 2b KEYNOTE-942 NCT03897881	~60% CD8^+^ and ~24% CD4^+^ T- cell responses	Weber et al. [8] Gainor et al. [24]
DNA	GNOS-PV02	nsSNVs, up to 40 neoantigens	IM	Advanced HCC	ICI	36 patients	Phase 1/2 study NCT04251117	Mostly CD8^+^ T-cell responses	Yarchoan et al. [27]
	VB10.NEO	nsSNVs, frameshifts up to 20 neoantigens	IM	Melanoma, NSCLC, SCCHN, RCC	ICI	41 patients	Phase 1 NCT05018273	Mostly CD8^+^ T -cell responses	Krauss et al. [31]
Viral	Heterologous ChAd68 and samRNA	nsSNVs, up to 20 neoantigens	IM	metastatic MSS-CRC, NSCLC or GEA	ICI	14 patients	Phase 1/2 study NCT03639714	CD8^+^ T-cell responses	Palmer et al. [32]
	Heterologous ChAd68 and samRNA	nsSNVs, up to 20 neoantigens	IM	metastatic MSS-CRC	ICI	104 patients	Phase 2/3 NCT05141721	Not published yet	NCT05141721

ChAd, chimpanzee adenovirus; CRC, colorectal cancer; GEA, gastroesophageal adenocarcinoma; HCC, hepatocellular carcinoma; ICI, immune checkpoint inhibitor; IM, intramuscular; IV, intravenous; LNP, lipid nanoparticle; LPX, lipoplex; MSS, microsatellite stable; MSS-CRC, microsatellite-stable colorectal cancer; NSCLC, non-small-cell lung cancer; nsSNVs, non-synonymous single-nucleotide variants; PDAC, pancreatic ductal adenocarcinoma; RCC, renal carcinoma; samRNA, self-amplifying mRNA; SCCHN, squamous cell carcinoma of head and neck.

Taken together, these early-phase studies demonstrate that personalised neoantigen vaccines can induce tumour-specific T-cell responses across diverse tumour types and delivery platforms [22,33]. However, because most trials were small-phase I studies not designed to assess efficacy, the durability and clinical relevance of these responses remain unclear. Even with multiple epitopes included, the magnitude and breadth of responses have been inconsistent, highlighting limitations in antigen selection, formulation, and delivery. These challenges underscore the importance of refining vaccine design and exploring antigen sources beyond canonical exonic mutations [34].

## 4. Identification, Prioritisation, and Immunogenicity of Neoantigens

Canonical neoantigens that arise from nsSNVs and INDELs in coding regions remain the most extensively studied and clinically advanced class of tumour-specific antigens [22], and, because these alterations generate amino-acid changes that are not present in healthy tissues, they bypass central tolerance and can be selectively recognised as non-self by T cells [reviewed in [35]]. Consequently, they form the backbone of most current neoantigen vaccine trials and personalised immunotherapy pipelines. In addition to SNVs and INDELs, gene fusion resulting from structural genomic alterations represents another clinically relevant source of canonical neoantigens [36].

Canonical neoantigens are typically identified through whole-exome and RNA sequencing of tumour–normal pairs, followed by in silico prediction of peptide–MHC class I or class II binding using increasingly refined computational algorithms [22,37,38,39]. To improve selection accuracy, predicted MHC-I binding affinity is often used as a primary filter, with peptides <500 nM, and especially <50 nM, preferentially selected. Additional features, including peptide–MHC stability, mutant allele expression, and the Differential Agretopicity Index (DAI), reflecting the predicted change in binding between mutant and wild-type sequences, further help to identify epitopes with increased “non-self” character and have been associated with improved survival in several tumour types [40,41,42]. Many modern pipelines now incorporate anchor residue changes, motif compatibility, and predicted TCR-contact residues to refine this prioritisation [43].

Immunopeptidomics provides a powerful tool to directly determine which peptides are processed and presented on tumour MHC molecules. Mass-spectrometry (MS)-based profiling can validate predicted epitopes, uncover peptides missed by computational algorithms, and quantify their relative abundance [44]. However, MS is inherently limited in sensitivity and does not capture all high-affinity predicted peptides [45], while many eluted ligands fail to elicit functional T-cell responses. Consistent with this, large-scale immunopeptidomic studies demonstrate that tumour-specific neoantigens constitute only a minute fraction of the MS-accessible MHC-I repertoire, rendering their reliable detection challenging even with substantial tumour input material [46,47]. As a result, proteogenomic frameworks that integrate genomic, transcriptomic, and immunopeptidomic data [47], as well as artificial intelligence [43,48] have become increasingly adopted for defining the canonical immunopeptidome and improving neoantigen selection [47].

Clonality of neoantigen expression in tumours is now a key consideration in neoantigen prioritisation. Early driver mutations typically give rise to clonal neoantigens present across all tumour cells, whereas later passenger events often generate subclonal epitopes confined to specific tumour subpopulations [49]. Clonality is inferred from bulk sequencing by integrating variant allele frequency, tumour purity, and copy-number state to estimate the cancer cell fraction, with tools such as PyClone [50] (https://github.com/Roth-Lab/pyclone; accessed on 15 January 2026) used to group mutations into clonal and subclonal clusters. Because clonal neoantigens are more consistently presented and more likely to sustain durable T-cell responses [16], clonality is increasingly used as key criterion in prioritising canonical neoantigens for vaccine development and T-cell-based therapies [16,51].

Gene fusions, resulting from fusion breakpoints in DNA, generate unique junctional peptides absent from the normal proteome, often producing highly immunogenic clonally expressed epitopes [36,52]. This source of antigen is becoming part of the canonical antigenic repertoire. Early studies demonstrated that fusion proteins such as BCR–ABL (CML) elicit autologous T-cell responses, establishing clinical feasibility [53]. Recent advances in long-read RNA sequencing, along with dedicated fusion-calling algorithms like GFvoter (https://github.com/xiaolan-z/GFvoter; accessed on 15 January 2026), FusionSeeker (https://github.com/Maggi-Chen/FusionSeeker; accessed on 15 January 2026), and JAFFAL (https://github.com/Oshlack/JAFFA/wiki; accessed on 15 January 2026), offer enhanced capacity to resolve full-length fusion transcripts and detect complex rearrangements with reduced artefacts [54,55,56]. Several clinical studies have already incorporated fusion-derived neoantigens into personalised vaccine platforms. More recently, personalised vaccine trials in glioma and paediatric solid tumours have included PAX3–FOXO1, EWS–FLI1, and CBFB–MYH11 fusion peptides as vaccine targets [reviewed in [22]].

## 5. Heterogeneity in Canonical Neoantigen Immunogenicity: Implications for Expanded Antigen Discovery

Despite major advances in genomic profiling, epitope prediction, and immunopeptidomics, the functional immunogenicity of canonical neoantigens remains highly variable across cancers and vaccine platforms. Clinical studies consistently show that only a subset of predicted nsSNV- and INDEL-derived epitopes elicit measurable T-cell responses, with per-epitope immunogenicity ranging from ~10% in low-mutation-burden or immunosuppressive tumours [7] to >60% in optimised vaccine platforms [11]. For example, the mRNA neoantigen cancer vaccine autogene cevumeran generated strong CD8^+^ T-cell responses but ex vivo reactivity to only ~11 of encoded epitopes in PDAC across all tested patients [7], whereas mRNA-4157 induced responses against ~23–30% of predicted neoantigens in non-small-cell lung cancer (NSCLC) and melanoma patients [24]. Higher per-epitope immunogenicity has been observed in melanoma peptide vaccines, with ~60% of long peptides eliciting CD4^+^ T-cell responses and ~16–20% of predicted class I epitopes generating CD8^+^ T-cell responses in the NeoVax trial, while further optimisation in NeoVax-MI increased detectable ex vivo responses to ~63% of immunising peptides [9,11,12].

Nonetheless, collectively, vaccine-induced CD8^+^ T-cell frequencies remain comparatively low (approximately between 0.1 and 0.5% of circulating cytotoxic lymphocytes when measured ex vivo) [9,12,13,30,57], in contrast to antiviral vaccines such as yellow fever, vaccinia, or SARS-CoV-2 mRNA vaccines, which can drive CD8^+^ T- cell expansions an order of magnitude higher [58,59]. This disparity is thought to reflect several factors that differ between tumour and viral antigens, including lower antigen abundance, weaker TCR affinity for many SNV/INDEL-derived peptides, and suboptimal activation of professional antigen-presenting cells in the tumour microenvironment [reviewed in [60]].

Taken together, these observations highlight that, although well-selected canonical neoantigens can elicit durable tumour-specific immunity and remain central to current personalised vaccine strategies, their overall number and immunogenic potential can be limited in many cancers. This has motivated parallel efforts to expand antigen discovery beyond nsSNVs and INDELs toward non-canonical antigen sources that may be more abundant, more consistently presented, or intrinsically more immunogenic.

## 6. Beyond Canonical Neoantigens: The “Dark Matter” of the Antigenome

### Conceptual Overview: Why Go Beyond the Exome?

The efficacy of ICI therapy demonstrates that potent antitumour immunity can be achieved when endogenous T cells recognise tumour-specific antigens [14]. In some cancers, responses to ICI correlate with TMB, which serves as a broad surrogate for the number of SNV-derived neoantigens. However, studies have shown that TMB is not a universal predictor of ICI efficacy [61]. A recent analysis revisiting the evidence for TMB as an ICI biomarker found that TMB–response associations largely disappeared after correcting for disease subtype and multiple testing, indicating that TMB is not a consistent predictor of benefit [62]. These findings do not diminish the value of SNV-derived neoantigens but indicate that additional antigen sources may also contribute to ICI responses, particularly in cancers with few coding mutations.

**The “dark matter” antigenome**. Because TMB does not consistently predict response to ICI, several studies have proposed the idea of ‘dark matter’ antigens—tumour-specific peptides that shape immune responses, even though their genomic and transcriptomic origins are difficult to pinpoint with standard pipelines [10]. The analogy to cosmological dark matter is apt: their presence is inferred through measurable immune effects despite their relative invisibility to conventional detection methods [63]. For more than a decade, neoantigen discovery has relied on exome or genome sequencing combined with peptide–MHC binding prediction, often validated by mass-spectrometry-based immunopeptidomics in annotated regions [44]. These canonical approaches have been invaluable in identifying epitopes derived from coding mutations, yet their dependence on annotated protein databases has left large regions of the non-coding genome unexplored [64].

Although less than 2% of the genome is formally annotated as protein-coding [65], multi-omic analyses reveal that up to 75% may be transcribed and, under oncogenic stress or inflammation, translated into short-lived peptides [66]. These non-canonical translation events arise through diverse mechanisms, including aberrant splicing, RNA editing, upstream open reading frame (uORF) initiation, and reactivation of normally silenced genomic elements such as endogenous retroviruses and transposable elements [66,67]. Studies have confirmed that such atypical transcriptional and translational processes can generate novel peptides with no overlap to the annotated proteome [17,68], revealing a hidden antigenic layer, the “dark matter”, which broadens the landscape of potential tumour-restricted epitopes.

**Figure 1:**
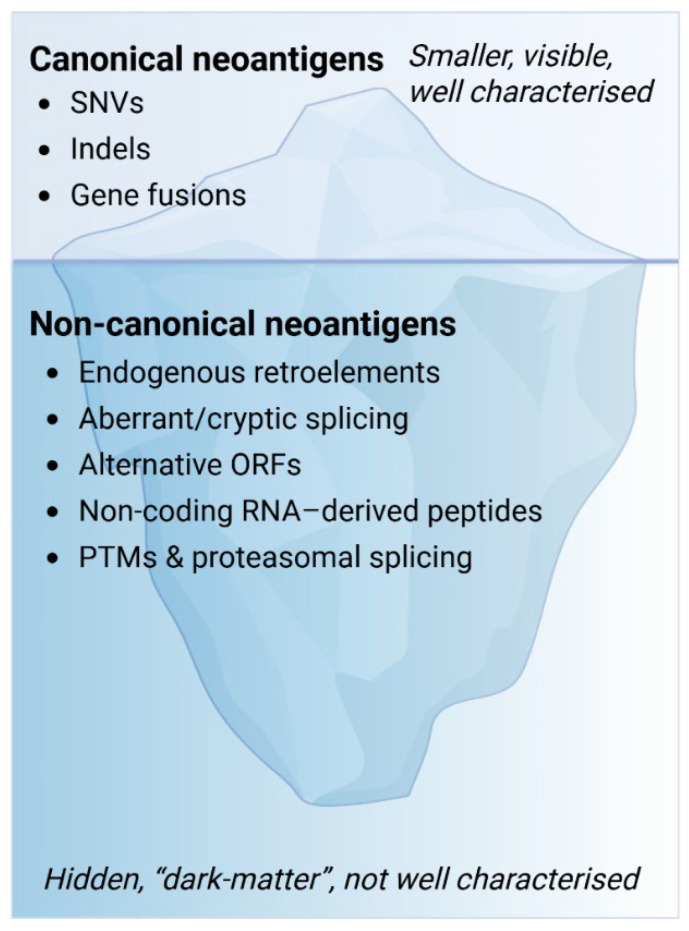
The tumour neoantigen landscape illustrated as an “iceberg model”. Canonical neoantigens arising from SNVs, indels, and gene fusions represent the visible “tip of the iceberg” and are generally well characterised. Beneath the surface lies a much larger pool of non-canonical neoantigens generated through dysregulation across the genome, transcriptome, and proteome. These include peptides derived from endogenous retroelements, aberrant splicing, non-coding RNAs, alternative open reading frames, and post-translational or proteasomal splicing events, collectively forming a substantial “dark matter” antigenic space that remains poorly characterised. Created in BioRender. Rwandamuriye, F.X. (2025) https://app.biorender.com/illustrations/692a6a776fe8c057bd7431a1 (accessed on 15 January 2026).

Thus, exome-centric views impose clear limitations, particularly for low-mutation tumours where few high-confidence candidates exist. Importantly, advances in mass-spectrometry-based immunopeptidomics have provided direct evidence that many of these non-canonical peptides are naturally processed and presented by HLA molecules [17,66]. These observations suggest that non-canonical translation products are not merely transcriptional artefacts but bona fide components of the tumour immunopeptidome. Consequently, canonical neoantigens may represent only the visible “tip of the iceberg,” while a much larger underexplored layer of non-canonical antigens lies concealed beneath (Figure 1).

## 7. Biological Sources of Non-Canonical Neoantigens

**Figure 2:**
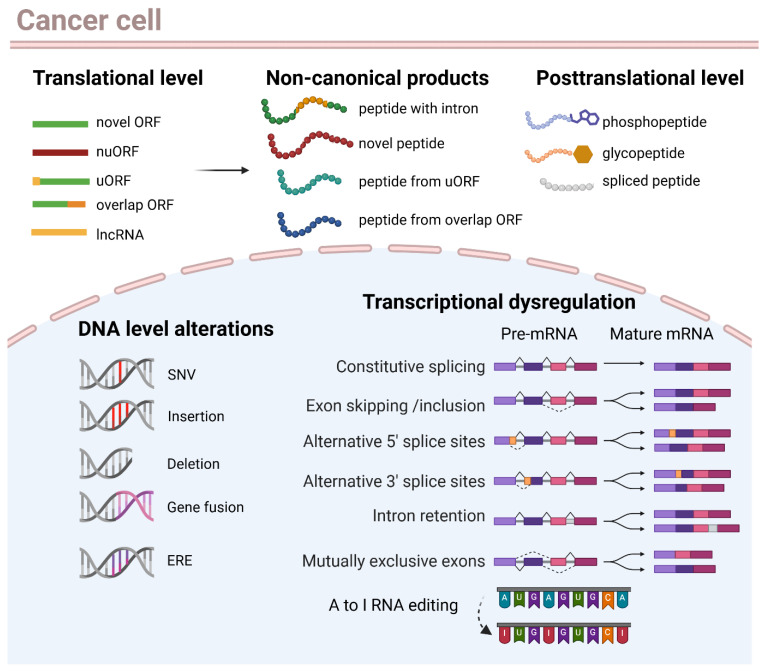
Source of canonical and non-canonical neoantigens. Canonical neoantigens can arise from genomic alterations such as single-nucleotide variants (SNVs), insertions and deletions (indels), and gene fusions that change protein-coding sequences. Non-canonical neoantigens emerge from broader dysregulation across the genome, transcriptome, and proteome. At the DNA level, these include activation of endogenous retroelements (EREs) and other genomic alterations. At the transcriptional level, non-canonical neoantigens are generated through aberrant splicing, including intron retention, exon skipping or inclusion, alternative 5’ and 3’ splice-site usage, and mutually exclusive exons, as well as RNA sequence modifications such as A-to-I RNA editing. At the translational level, alternative translation events give rise to peptides from long non-coding RNAs (lncRNAs), novel unannotated ORFs (nuORFs), upstream ORFs (uORFs), overlapping ORFs, and intron-derived sequences. At the post-translational level, additional antigenic diversity is introduced through post-translational modifications such as phosphorylation, glycosylation, and proteasomal peptide splicing. Created in BioRender Rwandamuriye, F.X. (2025) https://app.biorender.com/illustrations/692a6ad22d0744dc5df5f2d0 (accessed on 15 January 2026).

Non-canonical antigens arise from a spectrum of transcriptional, translational, and post-translational alterations (Figure 2). These include aberrant splicing and intron retention, alternative translation initiation, reactivation of transposable elements, and post-translational modifications [19,20,67].

### 7.1. Transcriptional Dysregulation: Alternative Splicing, Intron Retention, and RNA Editing

Splicing is the physiological process by which introns are removed from precursor mRNA and exons are ligated to generate mature transcripts. Alternative splicing is highly prevalent, affecting ~95% of human genes [69], and is normally tightly regulated because it shapes most cellular programs. In cancer, this regulation is frequently disrupted, producing tumour-biased isoforms that support proliferation, motility, and therapy resistance [70,71], and tumour-specific alternative splicing signatures have been linked to clinical outcomes and treatment response [72].

Splicing dysregulation arises through both cis- and trans-acting mechanisms (reviewed in detail [22]). Cis-acting alterations affecting splice sites or regulatory motifs drive exon skipping, intron retention, and alternative 5′/3′ splice-site usage [73,74]. Although intron-retained transcripts are typically degraded by nonsense-mediated mRNA decay (NMD) [75], antigenic peptides can still be generated during early translation before degradation [76,77,78]. Large-scale analyses from The Cancer Genome Atlas (TCGA) have identified numerous tumour-unique exon–exon junctions, consistent with a substantial pool of “neojunction”-derived neoantigens [79]. Trans-acting alterations further amplify splicing errors genome-wide. Somatic mutations in spliceosomal components such as SF3B1, U2AF1, SRSF2, and related factors are common in haematological malignancies [80] and occur across solid tumours, producing broad repertoires of aberrant transcripts with antigenic potential [81,82]. Notably, SF3B1-mutant uveal melanoma generates splice-derived neoepitopes recognised by CD8^+^ T cells [83]. Splicing fidelity can also be destabilised by epigenetic changes or post-translational modifications of splicing proteins [84].

Collectively, transcriptional dysregulation represents one of the richest sources of non-canonical epitopes, generating novel transcripts through aberrant splicing, intron retention, and cryptic transcription [17,85]. Immunopeptidomic analyses confirmed that these aberrant transcripts can yield HLA-I-presented epitopes in leukaemia, lymphoma, and solid tumours [17,18,85,86,87].

Additional layers of transcriptional diversification further expand this antigenic space. Adenosine-to-inosine (A-to-I) RNA editing catalysed by ADAR enzymes can recode codons, introducing amino-acid substitutions not present in the genome [88]. Edited peptides can exhibit altered MHC binding and tumour specificity, and dysregulated RNA editing is increasingly recognised in cancers such as glioblastoma and hepatocellular carcinoma [89]. Moreover, non-canonical splicing can join coding exons to transposable elements. In patients with NSCLC, Merlotti et al. showed that such junction-derived peptides are recurrently presented by HLA-I molecules and elicit CD8^+^ T-cell recognition in both tumours and tumour-draining lymph nodes [90].

### 7.2. Alternative Translation Products

Advances in genome-wide sequencing, immunopeptidomics, proteogenomics, and ribosome profiling have revealed that some regions previously considered as non-coding possess latent or context-dependent coding capacity [17]. Although the precise molecular mechanisms underlying cancer-selective non-canonical translation are incompletely understood, aberrant translation of non-coding transcripts is a recurrent feature in different cancers [17,18,67].

Cancer-associated dysregulation of translation, together with epigenetic reprogramming, can derepress or aberrantly activate non-coding regions, increasing the pool of RNA substrates accessible to ribosomal scanning and non-canonical translation [91,92]. This translational dysregulation can relax normal constraints on translation initiation, allowing ribosomes to initiate at alternative start sites rather than the canonical start codon [93]. As a result, peptides can be generated from regions upstream, within, or downstream of annotated coding regions, as well as from transcripts previously thought to be non-coding, such as small open reading frames, long non-coding RNAs (lncRNAs), circular RNAs (circRNAs), antisense RNAs, and pseudogenes [64,94,95].

Large-scale proteogenomic analyses have uncovered thousands of non-canonical peptides arising from these unannotated translation events. For instance, Ouspenskaia et al. identified numerous tumour-enriched peptides, including hundreds derived from lncRNAs, which are absent from healthy tissues, underscoring non-canonical translation as a rich source of tumour-specific antigens [67]. Complementing these findings, Ely et al. used high-depth immunopeptidomics in pancreatic cancer organoids and tumours to show that non-canonical antigens can originate from lncRNAs, untranslated regions, and alternative reading frames [18].

Oncogenic rewiring of translational control pathways, such as dysregulated mTOR–eIF4F signalling and altered eIF2α-dependent stress responses, can relax start-codon stringency and favour alternative or non-AUG initiation, thereby increasing the diversity of translated products in cancer cells [96,97]. Consistent with this altered translational landscape, ribosome profiling has directly confirmed widespread initiation from non-AUG codons, revealing widespread non-canonical initiation events producing MHC-presented peptides [17,21,98,99]. Nevertheless, although tumour-restricted presentation of alternative translation products has been demonstrated, the mechanisms that confer cancer specificity on these events remain incompletely understood.

### 7.3. Endogenous Retroviral and Transposable Elements

Epigenetic derepression of transposable elements (TEs) and endogenous retroviral elements (ERVs) generates a distinct pool of tumour-specific non-canonical antigens [100]. Under normal conditions, TEs and ERVs are tightly silenced through a combination of DNA methylation, repressive histone marks, and RNA-mediated repression; however, these regulatory layers are frequently disrupted in cancer, leading to their transcriptional reactivation [101]. These normally silenced sequences, when reactivated, can produce transcripts and proteins absent from healthy adult tissues but readily presented on tumour HLA molecules [102].

Mechanistically, reactivation of TEs in cancer is closely associated with global DNA hypomethylation, including recurrent demethylation of CpG sites within the LINE-1 5′UTR promoter across many tumour types [103]. However, locus-resolved analyses show that LINE-1 hypomethylation is heterogeneous and not always sufficient for expression, indicating that multiple redundant epigenetic silencing pathways normally restrain TE activity [104]. When these controls are disrupted, TEs can be co-opted through “onco-exaptation”, whereby TE-derived promoters or enhancers aberrantly activate proto-oncogenes or rewire tumour transcriptional programs [22,105]. Strong protein-level evidence supports widespread transposable element activation in cancer, with LINE-1 ORF1p detected in nearly half of human tumours, particularly high-grade cancers, while remaining absent from normal tissues [106]. In breast and epithelial cancers, elevated ORF1p/ORF2p expression correlates with aggressive disease, poor survival, and metastatic features and has enabled development of ultrasensitive plasma ORF1p assays as tumour-associated biomarkers [107].

Although ERV retrotransposition events are rare, the regulatory capacity of ERV sequences enables tumour-specific transcription and downstream peptide expression. For instance, in ovarian cancer, HERV-K/HML-2 antigens were shown to be expressed in 50% of tumours but not in normal ovarian tissues [108]. Immunopeptidomics confirmed their presentation on tumour cells, and patient-derived T cells could be expanded to recognise these epitopes. Importantly, HERV-specific CD8^+^ T cells selectively killed ovarian cancer cells in vitro and reduced tumour growth in vivo, demonstrating both tumour specificity and therapeutic potential [108].

More broadly, large-scale analyses have highlighted the clinical relevance of ERV expression in other cancers. Garde et al. demonstrated that the breadth of endogenous ERV expression stratified checkpoint inhibitor-treated melanoma patients into groups with distinct survival outcomes [109]. To systematically capture this repertoire, they developed ObsERV, a computational pipeline for predicting EVE-derived epitopes. Several ObsERV-predicted peptides were validated as being presented by HLA molecules and induced durable polyfunctional CD4^+^- and CD8^+^ T-cell responses, conferring long-term tumour protection in preclinical neoantigen vaccine models [109].

Collectively, with emerging evidence that retroelement dysregulation represents a broader hallmark of cancer [103,110], these findings establish TE and ERV reactivation as a biologically grounded, protein-level-validated, and clinically relevant source of non-canonical antigens.

### 7.4. Post-Translational Modifications (PTMs)

Post-translational modifications (PTMs) such as phosphorylation, glycosylation, citrullination, and proteasome-mediated splicing represent an additional mechanism generating non-canonical epitopes [111,112]. In cancer, PTMs arise from oncogenic signalling rewiring, metabolic reprogramming, and disrupted proteostasis, which collectively reshape the cellular modification landscape compared with normal tissues [113]. Aberrant kinase activity can create tumour-specific phosphopeptides detectable on MHC-I molecules in leukaemia and solid tumours, reflecting chronic hyperactivation of pathways such as MAPK, PI3K–AKT, and CDK signalling, increasing both the abundance and diversity of phosphorylated substrates available for antigen processing [111,114,115], while O-GlcNAc-modified peptides in leukaemia elicit multifunctional memory T-cell responses and selective cytotoxicity [116]. This enrichment of O-GlcNAcylated antigens is consistent with altered nutrient sensing and increased flux through the hexosamine biosynthetic pathway in malignant cells, particularly in haematological malignancies with low mutational burden [113].

Proteasome-mediated peptide splicing, although debated, has been experimentally validated as a source of immunogenic ligands [112,117]. Cancer-associated changes in proteasome composition, oxidative stress within the tumour microenvironment, and altered antigen-processing machinery can further bias peptide cleavage and splicing outcomes, enabling the generation of spliced epitopes that are rarely produced in normal cells [117]. In parallel, impaired Transporter Associated with Antigen Processing (TAP) or HLA-I function can expose a distinct class of unmutated T-cell epitopes associated with impaired peptide processing, which are selectively presented by antigen-processing-deficient tumour cells and escape central tolerance (reviewed in [22]). Together, PTM-derived epitopes highlight how cancer-specific disruption of signalling, metabolism, and protein homeostasis diversify the antigenic landscape in ways not seen in normal physiology.

Collectively, these mechanisms illustrate the extraordinary diversity of non-canonical neoantigens generated through genetic, transcriptional, translational, and post-translational alterations. Unlike canonical epitopes, many of these peptides are absent from healthy tissues, enriched in tumours, and capable of evading central tolerance, making them compelling targets for next-generation cancer vaccines and immunotherapies. However, their heterogeneity and often low abundance pose significant challenges for detection and validation. Realising the full therapeutic potential of these hidden antigens will depend on sensitive proteogenomic discovery pipelines [44,118], rigorous immunological characterisation, and rational vaccine design, as explored in the following sections.

## 8. Illuminating the Dark Antigenome: Computational and Proteogenomic Approaches to Identify Non-Canonical Antigens

The emergence of integrative proteogenomic and translatomic methods is providing a more complete view of the tumour antigen landscape and substantially expanding the detectable antigenic repertoire [66]. An early proof-of-concept study by Laumont et al. used RNA-seq integrated with customised mass-spectrometry searches to identify MHC-I-bound peptides derived from introns, untranslated regions (UTRs), and out-of-frame translation events [68]. Although performed in an Epstein–Barr virus-transformed B-cell line, the work offered the first direct evidence that non-canonical peptides can be naturally processed and presented by MHC molecules.

Subsequent studies extended these observations by incorporating direct evidence of translation [98,119] to distinguish genuine protein synthesis from background transcription. By combining ribosome profiling (Ribo-seq) with immunopeptidomics, these analyses demonstrated that many cryptic peptides originate from actively translated open reading frames rather than transcriptional noise [67,85].

More recently, large-scale analyses have shown that cryptic peptides are recurrent components of the tumour immunopeptidome across cancer types [120]. These conceptual advances necessitated the development of specialised computational pipelines capable of detecting non-canonical translation events, resolving their tumour specificity, and linking transcriptional aberrations to HLA-presented peptides.

## 9. Bioinformatics Tools for Identification of Non-Canonical Neoantigens

Bioinformatic analysis is central to the identification of non-canonical neoantigens and underpins interpretation of RNA sequencing, translatomic, and immunopeptidomic datasets. As detailed in recent reviews [118,121,122], computational approaches are indispensable for detecting aberrant transcriptional processes that give rise to non-canonical translation products. Accurate identification requires dedicated frameworks capable of detecting tumour-associated aberrations while accounting for biological variability, tumour heterogeneity, and technical noise.

## 10. Computational Tools to Detect Alternative Splicing-Derived Neoantigens

For alternative splicing events, two broad computational strategies are commonly applied to RNA-sequencing data: event-based detection of local splicing aberrations and reconstruction of full-length transcript isoforms [123]. Event-based approaches focus on localised changes in splice junction usage, exon inclusion, or intron retention without reconstructing complete transcripts. Widely used tools such as rMATS [124] (https://github.com/Xinglab/rmats-turbo; accessed on 15 January 2026) and MAJIQ (V1) [125] leverage spliced read alignments, guided but not constrained by reference annotations, to detect both annotated and unannotated splicing events and quantify differential usage between tumour and normal samples.

A key limitation of rMATS- and MAJIQ-like approaches is their optimisation for cohort-level comparisons, which can reduce sensitivity for rare patient-specific splicing events relevant to personalised immunotherapy [121]. To address this limitation, outlier-based frameworks have been developed for imbalanced tumour–normal analyses. Tools like Bisbee [126] apply a Bayesian beta-binomial model to identify splicing outliers relative to a reference panel of normal samples and have been benchmarked against protein-level evidence. FRASER (https://bioconductor.org/packages/release/bioc/html/FRASER.html; accessed on 15 January 2026) uses count-based statistical modelling with latent confounder correction to detect aberrant splicing and intron retention directly from alignment files, reducing false positives while maintaining sensitivity [127]. Despite these advances, event-based methods generally do not resolve transcript structure or reading frame, complicating downstream prediction of translated peptides.

Transcript reconstruction approaches aim to overcome this limitation by inferring complete isoforms prior to in silico translation. Reference-guided assemblers such as StringTie [128] (https://github.com/gpertea/stringtie; accessed on 15 January 2026) and Scallop [129] (https://github.com/Kingsford-Group/scallop; accessed on 15 January 2026) reconstruct transcripts by traversing splice graphs derived from short-read RNA-seq data. However, reconstruction accuracy is limited by short read length, exon sharing, and multi-mapping, particularly for low-abundance or complex isoforms (reviewed in [121]). Long-read RNA sequencing improves isoform resolution and enables direct observation of complex splicing patterns, but higher error rates necessitate specialised correction and polishing pipelines [130]. Commonly used approaches include Iso-Seq/ToFU, Scallop-LR, and hybrid short- and long-read strategies such as FLAIR, which leverage short-read accuracy to correct long-read errors [131,132,133]. While these methods improve ORF inference, they remain computationally demanding and may still miss transient or low-level tumour-specific transcripts.

## 11. Beyond Splicing Events: Other Non-Canonical Transcript Classes

Beyond splicing events, specialised tools have been developed to detect additional aberrant transcript classes. Intron retention can be quantified using IRFinder v1.3.1 [134], transposable element (TE)–derived transcripts using REdiscoverTE (https://github.com/ucsffrancislab/REdiscoverTE; accessed on 15 January 2026), which explicitly models repetitive mapping [102], and circular RNAs using CIRIquant v1.1.3 [135]. These transcript classes pose distinct computational challenges due to repetitive sequences, non-linear transcript structures or low expression levels.

Across all approaches, tumour specificity is typically inferred through comparison with matched normal tissue and expanded reference datasets such as GTEx [136] or TCGA [137], although inter-individual variability and incomplete normal tissue representation remain limitations, as discussed in the Section 16 of this review.

## 12. Integrated Proteogenomic and Translatomic Strategies

Because many cryptic peptides arise from translation products absent from canonical proteomes, their identification commonly relies on customised RNA-seq-derived protein databases. Integrated proteogenomic and translatomic strategies are increasingly used to provide orthogonal evidence of active translation and antigen presentation [66]. However, in silico three- or six-frame translation substantially expands the search space, increasing computational burden and false discovery rates [128,138].

To mitigate this hurdle, several filtering strategies have been developed. Tumour–normal subtraction workflows retain tumour-specific RNA subsequences for translation and MS database construction [17]. For TE-derived antigens, tools such as REdiscoverTE (https://github.com/ucsffrancislab/REdiscoverTE; accessed on 15 January 2026), enable targeted peptide library generation [102], while peptide-centric strategies such as Peptide-PRISM (https://erhard-lab.de/software; accessed on 15 January 2026) combine de novo MS sequencing with genome-wide translated databases to identify cryptic MHC-I ligands [19], although these approaches remain computationally intensive and require extensive validation [121].

Ribosome profiling (Ribo-seq) directly captures actively translated regions, enabling the generation of refined reference proteomes with constrained ORFs and substantially reduced search space. Computational tools such as ribotricer [139] (https://github.com/smithlabcode/ribotricer; accessed on 15 January 2026), RiboHMM [140] (https://github.com/rajanil/riboHMM; accessed on 15 January 2026), RibORF [141] (https://github.com/zhejilab/RibORF; accessed on 15 January 2026), and PRICE [142] (https://github.com/erhard-lab/price; accessed on 15 January 2026) detect translated ORFs by exploiting ribosomal three-nucleotide periodicity, hidden Markov models, support vector machines, or expectation–maximisation frameworks, respectively. Incorporation of Ribo-seq-derived ORFs into proteogenomic pipelines has enabled robust identification of peptides originating from unannotated ORFs located in UTRs, internal coding regions, and long non-coding RNAs [17,66,67].

In summary, rigorous integration of RNA-seq, Ribo-seq, and immunopeptidomic data, together with advanced bioinformatic tools and stringent quality-control frameworks, is essential for prioritising biologically meaningful and therapeutically actionable cryptic antigens.

## 13. Prevalence and Biological Significance

Accumulating evidence indicates that non-canonical peptides are not isolated events but form a quantitatively significant fraction of the tumour immunopeptidome [17]. Multi-omic and immunopeptidomic studies have revealed that non-canonical peptides are recurrent and biologically relevant components of the tumour immunopeptidome. Across cancer types, thousands of HLA-bound peptides map to non-coding, out-of-frame, or intergenic regions, indicating that antigen production extends well beyond annotated exons [17,68,143]. Although the precise estimates vary between analytical pipelines, the overall conclusion is consistent: cryptic translation is widespread and cannot be captured by exome-based discovery alone [120,143]. However, while these studies establish the prevalence of cryptic translation, the central question is whether these peptides are naturally processed and presented on tumour MHC molecules, enabling immune recognition [144].

Immunopeptidomic profiling across species provides quantitative evidence that non-canonical peptides are naturally presented on MHC molecules. In murine models, Laumont et al. reported that up to 60% of MHC-I-associated ligands arose from intronic, UTR, or out-of-frame regions rather than annotated exons [17]. Human datasets show a similar pattern: Abelin et al. found that 30–50% of HLA-I peptides lacked canonical annotation [143], and, in a recent integrated analysis of melanoma and NSCLC human samples, Apavaloaei et al. showed that ~99% of tumour antigens originated from unmutated genomic regions [45]. Surprisingly, even in these high-tumour mutational burden cancers, mutation-derived tumour-specific antigens represented only ~1% of all detected HLA-I ligands. In contrast, aberrantly expressed tumour-specific antigens from intronic, UTR, pseudogene, or out-of-frame regions were far more abundant and strongly tumour-restricted, with 72% in melanoma and 88% in NSCLC showing no evidence of presentation in normal tissues [45]. Collectively, these findings confirm that non-canonical peptides constitute a substantial component of the tumour immunopeptidome. The next critical question is which of these presented ligands are truly immunogenic and capable of contributing meaningfully to antitumour immunity.

## 14. Functional Immunogenicity and Clinical Relevance

The key question for any candidate neoantigen is not merely whether it can be detected but whether it can elicit a productive T-cell response [35]. Despite studies showing the high abundance of non-canonical peptides in cancer cells, we do not have enough data to validate the role of these cryptic peptides in spontaneous or treatment-induced antitumour immunity in vivo. However, recent work has begun to shift the field from descriptive cataloguing toward mechanistic validation, helping to define which non-canonical peptides are genuinely immunogenic and what biological constraints shape their therapeutic potential.

### 14.1. Evidence of Immunogenicity in Murine Models

Using a proteogenomic approach, Laumont et al. identified 40 tumour-specific antigens in CT26 and EL4 murine models, approximately 90% of which originated from introns, UTRs, pseudogenes, or out-of-frame exons [17]. Vaccination with individual peptides representing these cryptic antigens showed heterogeneous protection (0–100%), and only highly expressed clonally distributed antigens generated robust CD8^+^ T-cell responses capable of durable tumour control [17]. These findings align with human data in which clonal neoantigen burden correlates with T-cell immunogenicity and clinical sensitivity to ICI therapy [16]. These results establish that non-canonical peptides can be strongly immunogenic, but effective responses may depend on expression level as well as clonality.

Building on these discoveries, Barczak et al. integrated transcriptomic, translatomic, and immunopeptidomic analyses in murine (CT26) and human (HCT116) colorectal cancer models, identifying hundreds of lncRNA-derived peptides (6.5% of MHC-I ligands in CT26) arising following epigenetic derepression [145]. Inhibition of PRMT5, a histone methyltransferase that constrains E2F1-mediated transcription, triggered activation and translation of lncRNAs encoding these peptides. Vaccination with viral vectors expressing twenty of the most abundant lncRNA-derived antigens elicited robust CD8^+^ T-cell responses and delayed tumour growth, demonstrating that cryptic translation from the non-coding genome can yield protective epitopes [145].

In ovarian cancer, Raja et al. reported that approximately 70% of prioritised lncRNA- and pseudogene-derived peptides induced polyfunctional CD8^+^ T-cell responses in autologous assays despite constituting <1% of the total ligandome [20], demonstrating that even low-abundance non-canonical peptides can generate biologically meaningful immunity.

Taken together, these studies spanning murine models and human tumours show that non-canonical translation products are not only detectable but can also be naturally presented and, in selected cases, immunogenic. However, most analyses to date have focused on individual tumour types or limited sample sets. This leaves a key unresolved question for the field: how broadly are non-canonical peptides presented across human cancers, and to what extent do they contribute to functional antitumour immunity?

### 14.2. Large-Scale Human Studies: Immunogenicity and Clinical Relevance

Following the demonstration that non-canonical peptides are widely presented across tumours, the next challenge is determining which of these antigens are functionally relevant in humans. Recent large-scale proteogenomic studies have begun to address this question by moving beyond cataloguing prevalence to directly testing immunogenicity and clinical relevance.

Apavaloaei et al. performed an integrated immunopeptidomic and transcriptomic analysis in melanoma and NSCLC and identified a set of aberrantly expressed tumour-specific antigens that were absent from normal immunopeptidomes, with 72% and 88% showing tumour-restricted expression, respectively [45]. Using curated normal-tissue immunopeptidome references to exclude peptides detectable in benign tissues, they found that most tumour-presented antigens were unmutated. Neoantigens originating from somatic mutations represented only a very small fraction (~1%) of all the HLA-I-associated peptides identified. Notably, functional testing with matched PBMCs and TILs showed that eight out of the sixty-two evaluated non-canonical peptides could elicit immune reactivity. Importantly, in patients treated with anti–PD-1/PD-L1, T cells specific to aberrantly expressed tumour-specific antigens expanded after therapy, and some of these antigens were lost or downregulated in responding tumours, consistent with immune-mediated editing [146]. These data suggest that non-canonical unmutated tumour-specific antigens can participate in ICI-driven immune responses, complementing classical SNV-derived neoantigens, even though overall cryptic neoantigen load did not correlate with ICI response. This observation, consistent with recent meta-analyses [62], suggests a threshold effect: the presence of one or a few immunogenic epitopes can be sufficient to support effective antitumour immunity, and additional epitopes confer little incremental benefit. Overall, clinical responses to checkpoint blockade in cancers with low TMB support the possibility that unmutated non-canonical tumour-specific antigens complement classical SNV-derived neoantigens in driving therapeutic benefit.

Lozano-Rabellá et al. extended these findings by identifying several hundred non-canonical peptides presented on HLA-I molecules of autologous tumour-derived cell lines from patients with melanoma and colorectal cancer. Functional assays using patient-matched PBMCs and TILs showed that approximately 10–15% of tested peptides elicited interferon gamma (IFN-γ) responses, confirming that a subset of cryptic peptides are both naturally presented and immunogenic [147]. Although presentation was confirmed on tumour-derived cells rather than primary biopsies, the study provides evidence that non-canonical peptides can be recognised by patient T cells.

In parallel, Schwarz et al. examined canonical and non-canonical antigen presentation in microsatellite-instable colorectal cancer (MSI-H CRC) using a combined immunopeptidomic and functional T-cell assay approach [148]. Across patient-derived CRC cell lines, they identified approximately 9000 canonical and ~130 cryptic HLA-I peptides, including 97 non-canonical cryptic peptides and three mutation-derived neoepitopes. Although cryptic peptides represented only ~1% of the total ligandome, several were naturally presented and able to prime or restimulate CD8^+^ T cells. Peptide-stimulated T cells produced IFN-γ, expressed CD107a, and could kill autologous tumour cells, indicating preserved cytotoxic potential [148].

Transposable element (TE) activation has been associated with immune infiltration and increased antigenicity in several tumour types [100,102]. Building on this concept, Merlotti et al. characterised TE-derived exon–transposon junctions (JETs) in >1000 TCGA NSCLC transcriptomes, identifying nearly 9000 JETs, of which ~5% were tumour-restricted (tsJETs). Immunopeptidomic profiling confirmed 114 JET-derived peptides naturally presented on tumour HLA-I molecules, including several recurrent across patients [90]. Functional testing of 29 HLA-A*02:01-restricted peptides in blood from healthy donors demonstrated robust MHC binding and the capacity to expand peptide-specific CD8^+^ T cells, with induced IFN-γ and granzyme B responses and, in some cases, tumour-cell lysis in vitro. These findings highlight TE-driven exon fusions as a recurrent and immunogenic source of non-canonical tumour epitopes.

Collectively, these studies demonstrate that non-canonical epitopes can be recurrently presented, tumour-restricted, and functionally immunogenic (Table 2). When integrated with larger proteogenomic datasets reporting broad presentation but limited T-cell recognition, they suggest a hierarchical model: only a subset of cryptic peptides achieve potent high-quality immune visibility, often through stable expression or fusion with repetitive-element sequences. However, despite these encouraging findings, an important question remains: how reproducible and stable are these non-canonical antigens across time and tumour evolution? Unlike mutation-derived epitopes, which are fixed in the genome, hidden neoantigens often arise from dynamic processes such as aberrant splicing, transcriptional noise, or transposable element activation. Their presentation may therefore fluctuate, raising concerns about consistency and therapeutic reliability, factors that should be taken into consideration for vaccine design.

## 15. Implications of Non-Canonical Neoantigens on Vaccine Development

The discovery of hidden neoantigens reshapes the conceptual and practical foundations of cancer vaccine design. Incorporating non-canonical peptides expands the available epitope repertoire and enables multi-epitope constructs that combine canonical and cryptic targets, reducing the risk of immune escape. Importantly, pre-clinical and clinical evidence shows that cryptic peptides are naturally presented by HLA molecules and can drive potent CD8^+^ T-cell responses with tumour-selective cytotoxicity [17,90], fulfilling key criteria for vaccine translation.

One particularly appealing aspect of non-canonical antigens is that some are shared, or “public,” within tumour types. These recurrent cryptic epitopes raise the possibility of developing off-the-shelf vaccine formulations while still allowing patient-specific cryptic antigens to be incorporated into personalised vaccines. However, translating these antigens into routine clinical practice will require more reliable discovery pipelines, stronger evidence of tumour restriction, and careful safety evaluation.

## 16. Key Challenges and Considerations

**Identification and prioritisation**. A major methodological challenge in advancing non-canonical neoantigen discovery is that most current pipelines remain anchored to annotated coding regions. Recent integrative approaches combining mass-spectrometry-based immunopeptidomics, RNA-seq, and ribosome profiling have begun to address this limitation [66], but these methods remain technically demanding. Their implementation requires specialised mass-spectrometry platforms, extensive computational infrastructure, and substantial analytical expertise, which limits their use to a small number of researchers. As a result, systematic detection of cryptic antigens is still not feasible at scale, and most discovery pipelines continue to rely on exome-based prediction alone.

In addition to lacking infrastructure, prioritisation of the non-canonical peptides requires distinguishing identified peptides from those that are reliably presented and relevant to antitumour immunity. Three practical features can be used to guide this process. First, clonality and abundance have emerged as important considerations for vaccine design involving canonical neoantigens [17,49,50]. However, in the context of non-canonical neoantigens, these peptides are generated through highly variable or stress-responsive transcription [149] and are unlikely to be expressed or presented consistently across the tumour or timepoints, limiting their suitability as vaccine targets. Second, their tumour restriction must be clearly demonstrated for safety. Several groups now compare candidate peptides against benign tissue immunopeptidome atlases [45,143] to exclude cryptic ligands that are detectable in normal tissues or developmentally regulated compartments. This step is particularly important for antigens originating outside annotated exons, where physiological expression patterns are often poorly characterised. Third, some indication of functional relevance is needed. This does not imply that neoantigens must elicit spontaneous T-cell responses in vivo, and this is not a requirement for vaccine inclusion. Rather, minimal in vitro evidence of T-cell recognition helps to confirm that a peptide can indeed engage the human T-cell repertoire once it is presented at a sufficient density. Studies by Apavaloaei et al. and Lozano-Rabellá et al. highlight this distinction clearly: although mass spectrometry detected numerous cryptic peptides, only a minority demonstrated immunogenicity or multimer binding with autologous lymphocytes [45,147]. Developing rigorous prioritisation frameworks that incorporate these criteria will be essential before non-canonical antigens can be confidently integrated into personalised vaccine platforms or off-the-shelf immunotherapies.

**Reproducibility, stability, and implications for immunogenicity.** A key translational challenge is whether non-canonical antigens are reproducibly expressed and stably presented. While most mutation-derived epitopes are typically clonal and genetically fixed [150], many cryptic peptides arise from epigenetically plastic processes such as aberrant splicing or alternative translation [17,67]. These events fluctuate between cells and over time, resulting in subclonal or transient antigen expression [85]. For example, in mismatch-repair-deficient colorectal cancer, intron-retention-derived epitopes were shown to display a highly heterogeneous presentation, consistent with the underlying transcriptional instability of these tumours [151]. Likewise, integrated proteogenomic analyses indicate that many cryptic open reading frames (ORFs) are inducible under cellular stress but fall silent under homeostatic conditions [66,67]. These findings emphasise that a substantial fraction of non-canonical antigens are transient rather than constitutively expressed, reinforcing the need to prioritise only those peptides that show stable reproducible presentation.

This biological plasticity raises an important interpretative issue: Does failure to detect an aberrantly expressed tumour-specific antigen at a later timepoint distinguish between true antigen loss due to immune pressure and simple absence caused by the inherent instability of cryptic translation? For instance, in the Apavaloaei study [45], several T-cell clones specific to aberrantly expressed tumour-specific antigens expanded following ICI therapy, strongly implying that these epitopes were indeed presented and immunologically active in vivo. Yet, when the tumour samples were re-analysed after treatment, some of the corresponding peptides were no longer detectable [45]. This finding can be read in two ways: (i) immune-mediated antigen loss, consistent with immunoediting [152], where successful T-cell killing selects against antigen-expressing clones; or (ii) intrinsic instability, where transient cryptic ORFs are not consistently expressed at levels detectable by mass spectrometry.

**Tumour specificity and safety**. A central requirement for translating non-canonical neoantigens into therapeutic targets is clear evidence of tumour-restricted presentation. Many cryptic peptides originate from genomic regions that are epigenetically silenced or translationally repressed in healthy tissues, leading to their preferential expression in malignant cells [17,18]. This tumour enrichment makes them conceptually attractive candidates for vaccination. However, large pan-cancer immunopeptidomic datasets show that not all cryptic peptides are uniformly tumour-exclusive; some can occasionally be detected at low levels in benign tissues [45].

In practice, tumour specificity is typically inferred by comparing tumour expression with matched normal tissues. However, many cryptic peptides arise from epigenetically plastic transcriptional and translational processes that can fluctuate between cells and patients over time, resulting in transient, heterogeneous, or subclonal antigen expression. This intrinsic variability complicates the distinction between true tumour-restricted antigens and low-level background expression. Given these limitations, reliable assessment of tumour specificity requires comparisons that extend beyond tissue-matched controls alone [67]. Large reference datasets incorporating unmatched donors and diverse tissue types, such as The Genotype-Tissue Expression (GTEx) project [136] and TCGA [137], are therefore commonly used to contextualise tumour-associated expression [67]. While these expanded comparisons improve sensitivity, they remain inherently incomplete as certain normal tissue subtypes are underrepresented, and baseline expression of aberrant transcripts can be patient-specific [121]. Furthermore, increasing stringency through broad normal-tissue filtering can inadvertently exclude biologically relevant targets due to technical noise, tumour-in-normal contamination, or low-level aberrant expression in non-malignant contexts [121].

Tumour specificity can be further assessed using direct evidence of HLA peptide presentation [118]. Public immunopeptidomic resources from healthy tissues, including large-scale HLA ligand atlases [153,154], provide critical references for excluding peptides presented outside malignant contexts. Machine-learning-based prioritisation trained on tumour and normal immunopeptidomes further refines candidate selection by identifying consistently tumour-enriched peptides [118]. However, computational prediction of immunogenicity remains limited, with models such as NetTepi v1.0 [155], PRIME v2.1 [156], and NetMHCpan-4.0-based predictors [157] offering only modest improvements over random ranking, reinforcing the need for experimental validation.

In summary, non-canonical neoantigens offer meaningful opportunities to strengthen cancer vaccine design by expanding the pool of targetable antigens. However, their biological variability, heterogeneous expression, and incomplete tumour specificity present clear challenges that necessitate rigorous prioritisation and validation. Ensuring that only reproducible, tumour-restricted, and immunologically actionable candidates progress into vaccine development will be critical for realising the therapeutic potential of this expanding antigen class. As discovery pipelines continue to integrate proteogenomic, translatomic, and immunological filtering, it will be feasible to define cryptic peptides that meet these translational criteria [45,85,90].

## 17. Outlook and Future Directions

Recent work on non-canonical neoantigens has reframed our understanding of the tumour immunopeptidome. Rather than representing a fixed list of mutation-derived peptides, tumour antigen presentation is now recognised as a dynamic process shaped by transcriptional, epigenetic, and translational plasticity. Despite these conceptual advances, sensitive and accurate identification of non-canonical tumour antigens remains challenging [118]. Moving this field towards clinical application will require continued development of proteogenomic pipelines and tighter integration of mechanistic insights with technological innovation, enabling more accurate and reliable identification of cryptic antigens.

**Integrating multi-omics and computational inference**. Multi-omic approaches that combine HLA ligand profiling with ribosome profiling, single-cell sequencing, and epigenomic datasets are beginning to define how cryptic antigen expression varies with tumour evolution, immune pressure, or treatment [45,66]. These integrative datasets provide complementary information on antigen abundance and context-dependent expression, helping to identify which non-canonical peptides are more likely to be consistently presented. In particular, single-cell RNA-seq has the potential to resolve intratumour heterogeneity and identify antigen source genes expressed only in restricted tumour subpopulations, enabling their inclusion in customised proteogenomic databases for downstream immunopeptidomic analysis [66]. Advances in computational modelling, including machine-learning methods trained on harmonised multi-omic datasets, will improve the prediction of peptide binding and presentation by HLA molecules and immunogenic potential [158], which are important for clinical relevance of non-canonical neoantigens.

## 18. Standardisation, Shared Resources, and Reproducibility

Progress in defining non-canonical neoantigens has accelerated with advances in immunopeptidomics [44,159], high-resolution sequencing, and integrative proteogenomic workflows [66]. However, in contrast to canonical neoantigen discovery, where mutation-calling pipelines and reporting standards are now well established [160], non-canonical antigen identification still suffers from considerable methodological variability. Differences in mass-spectrometry acquisition settings, ORF annotation, and peptide-calling thresholds can produce markedly different ligand sets from similar tissues. Such heterogeneity complicates cross-study comparison and limits independent verification of candidate antigens.

As occurred in the early evolution of genomic neoantigen prediction, the field would benefit from harmonised reporting guidelines covering sample processing, MS acquisition parameters, data normalisation, and criteria for peptide identification and false-discovery rate control. The Tumour Neoantigen Selection Alliance (TESLA) emphasised the value of such standardisation, showing that variability in analytical pipelines substantially affects peptide detection and prioritisation, and that shared benchmarks improve the robustness of neoantigen prediction across centres [160].

Developing shared reference datasets, particularly paired tumour–normal tissue immunopeptidomes generated under uniform conditions, would enable meaningful benchmarking of emerging proteogenomic pipelines and increase confidence that reported non-canonical peptides represent true biological presentation rather than analytical artefact. Additionally, public multi-omic repositories that integrate transcriptomic, MS-based immunopeptidomic, and ORF-level annotation data [161] will be essential, both for reproducibility and enabling groups without specialised mass-spectrometry infrastructure to participate in cryptic antigen discovery.

If these barriers can be overcome, non-canonical antigens may transform cancer immunotherapy by supplying new tumour-exclusive targets for both personalised and shared vaccine platforms. Their integration into neoantigen cancer vaccines may enable broader, more durable immune protection, especially in low-mutation-burden cancers where canonical neoantigens are scarce [36]. Early works show the feasibility of shared neoantigens [162]. Looking further ahead, real-time monitoring of antigen expression dynamics (via liquid biopsies or serial tumour/immune sampling) may enable adaptive vaccine design, responding to antigen loss or tumour evolution in real time [163].

## 19. Conclusions

The recognition that most presented tumour antigens originate beyond canonical coding regions could redefine the landscape of cancer immunology. Non-canonical peptides constitute a vast and previously underestimated source of tumour specificity.

Technological advances in high-resolution immunopeptidomics, ribosome profiling, and integrated computational analysis are now rapidly transforming what was once “dark matter” into a quantifiable and therapeutically tractable entity. For instance, recent pan-cancer peptide atlases demonstrate comparable presentation levels of canonical and non-canonical peptides [164], although demonstrating tumour-restricted presentation and the presence of functional antigen-specific T-cell responses remain critical considerations. In practical terms, successful translation will depend on combining multi-omic profiling, AI-based prediction, and robust functional validation within shared well-annotated datasets. If these elements can be integrated, carefully selected non-canonical neoantigens could become routine components of both personalised and off-the-shelf cancer vaccines.

## Figures and Tables

**Table 2:** Key studies on hidden neoantigens: methodologies and level of evidence.

Study Ref.	Tumour Type/Model	Non-Canonical	Main Approach	Immunological Validation	Key Findings
Laumont et al. [17]	Murine (CT26 and EL4), human samples	Introns, UTRs, pseudogenes	Bioinformatic + MS	ELISpot, tetramers, tumour rejection	60% of immunogenic neoepitopes from non-canonical regions. Strong CD8^+^ T-cell responses; heterogeneous. High expression + clonal distribution linked to durable control.
Barczak et al. [145]	Murine (CT26) and human (HCT116)	Small ORF in lncRNAs	Transcriptomic + translatomic + immunopeptidomics	Ex vivo–loaded DCs; ChAdOx1/MVA viral vector vaccination	lncRNA-derived peptides induced strong CD8^+^ T-cell responses and delayed tumour growth. 20 cryptic peptides elicited potent immune responses
Raja et al. [20]	Human cervical cancer	lncRNAs and pseudogenes	Immunopeptidomics + in silico prioritisation	Autologous T-cell assays	70% of prioritised non-canonical peptides induced polyfunctional CD8^+^ T-cell responses.
Apavaloaei et al. [45]	Human melanoma, NSCLC	Broad non-canonical regions	RNA-seq + MS	Autologous PBMC and TIL assays	99% of detected peptides were non-canonical; 72–88% tumour-restricted. 8/62 (~13%) induced high-avidity cytotoxic CD8^+^ T-cell responses.
Lozano-Rabellá et al. [147]	Melanoma, gynecologic, and head and neck cancer	5’UTR, off-frame, and ncRNA	RNA-seq/LC/MS-MS	Donor PBMC and TIL functional assays	~10–15% of non-canonical peptides tested elicited IFN-γ responses, 10% of non-canonical. Peptides were shared across at least two patients. In vitro stimulation identified T cells recognising three non-canonical peptides
Ely et al. [18]	Human pancreatic cancer organoids + bulk tumours	lncRNAs, 5′ or 3′ UTRs, alternative ORFs	proteogenomic and high-depth immunopeptidomics	ex vivo T-cell priming, expansion, tetramers, killing assays	>1000 cryptic peptides identified; ~30% cancer-restricted (CR); ~50% shared across patients; 36.3% CR peptides immunogenic (vs. 8.7% non-CR); TCRs isolated at single-cell resolution; CR-specific T cells killed pancreatic organoids ex vivo and in vivo.
Schwarz et al. [148]	microsatellite-instable colorectal cancer	Non-canonical ORFs	Immunopeptidomics	IFNγ ELISPot, Immunophenotyping	Non-canonical peptides ~1% of ligandome but several naturally presented and able to prime/restimulate CD8^+^ T cells.
Merlotti et al. [90]	NSCLC (TCGA transcriptomes + tumour samples)	JETs (junction-encoded transcripts)	Transcriptomics + immunopeptidomics	CD8^+^ T-cell expansion, IFN-γ, granzyme B, tumour lysis	~9000 JETs; ~5% tumour-restricted; 114 peptides naturally presented; 29 HLA-A*02:01 peptides induced strong CD8^+^ expansion and cytotoxicity; detectable also in healthy donors.

APC, antigen-presenting cell; ChAdOx1, chimpanzee adenovirus Oxford 1; CR: cancer-restricted DC, dendritic cell; ELISpot, enzyme-linked immunospot assay; HLA, human leukocyte antigen; ICS, intracellular cytokine staining; IFN-γ, interferon-gamma; JETs, junction-encoded transcripts; lncRNA, long non-coding RNA; MHC-I, major histocompatibility complex class I; MS, mass spectrometry; ncRNA, non-coding RNA; nsSNVs, non-synonymous single-nucleotide variants; ORF, open reading frame; PBMC, peripheral blood mononuclear cell; Ribo-seq, ribosome profiling; RNA-seq, RNA sequencing; TCR, T-cell receptor; TIL, tumour-infiltrating lymphocyte; UTR, untranslated region.

## Data Availability

No new data were created.
